# Overexpression of *TaWRKY146* Increases Drought Tolerance through Inducing Stomatal Closure in *Arabidopsis thaliana*

**DOI:** 10.3389/fpls.2017.02036

**Published:** 2017-11-24

**Authors:** Jianhui Ma, Xiaolong Gao, Qing Liu, Yun Shao, Daijing Zhang, Lina Jiang, Chunxi Li

**Affiliations:** College of Life Sciences, Henan Normal University, Xinxiang, China

**Keywords:** *Triticum aestivum* L., WRKY transcription factor, evolutionary analysis, drought tolerance, stomatal aperture

## Abstract

As a superfamily of transcription factors, the tryptophan-arginine-lysine-tyrosine (WRKY) transcription factors have been found to be essential for abiotic and biotic stress responses in plants. Currently, only 76 WRKY transcription factors in wheat could be identified in the NCBI database, among which only a few have been functionally analyzed. Herein, a total of 188 WRKY transcription factors were identified from the wheat genome database, which included 123 full-length coding sequences, and all of them were used for detailed evolution studies. By bioinformatics analysis, a WRKY transcription factor, named *TaWRKY146*, was found to be the homologous gene of *AtWRKY46*, overexpression of which leads to hypersensitivity to drought and salt stress in *Arabidopsis*. Consequently, the full length of *TaWRKY146* was cloned, and the expression levels of *TaWRKY146* were found significantly up-regulated in the leaves and roots of wheat seedlings, which were subjected to osmotic stress. Overexpression of *TaWRKY146* in *Arabidopsis* was shown to enhance drought tolerance by the induction of stomatal closure that reduced the transpiration rate. All these results provide a firm foundation for further identification of WRKY transcription factors with important functions in wheat.

## Introduction

Tryptophan-arginine-lysine-tyrosine (WRKY) transcription factors are one of the largest families of transcriptional regulators in plants. The WRKY proteins contain one or two conserved WRKY domains of about 60 amino acids. Based on their features, the WRKY transcription factors are divided into three main groups. Group I contains two WRKY domains and a C_2_H_2_ (CX_4-5_CX_22-23_HX_1_H) zinc finger motif, Group II contains one WRKY domain and a C_2_H_2_ zinc finger motif, and Group III contains one WRKY domain and a C_2_HC (CX_7_CX_23_HX_1_C) zinc finger motif (Ma et al., 2015). Since the identification of the first WRKY transcription factor in sweet potato (Ishiguro and Nakamura, 1994), a large number of WRKY transcription factors have been identified in different plants, several of which were reported to be involved in the responses to abiotic and biotic stress and in phytohormone-mediated signal transduction in plants.

In *Arabidopsis*, 61 WRKY transcription factors (AtWRKYs) were first identified by Eulgem et al. (2000), and were named sequentially from *AtWRKY1* to *AtWRKY61*. Yu et al. (2001) cloned *AtWRKY62*, and demonstrated that it specifically recognizes and binds to the promoter region of *NPR1*, thereby, positively modulating the transcription during the defense responses of plants. Dong et al. (2003) analyzed the genome database of *Arabidopsis* and identified 72 WRKY transcription factors, including the 62 previously identified AtWRKYs. In rice, 81 WRKY transcription factors (OsWRKYs) were first identified by Xie et al. (2005), among which the positive and negative expressed OsWRKYs by abscisic acid (ABA) signaling were determined in the aleurone cells. Ross et al. (2007) identified 98 and 102 WRKY transcription factors from the genome databases of *japonica* and *indica* rice, respectively, and studied the evolutionary history of these OsWRKYs in detail. On the basis of previous researches, Ramamoorthy et al. (2008) identified 103 WRKY transcription factors from the *japonica* genome database and determined their expression patterns under different abiotic and phytohormone treatments. With the publication of genome databases of different plants, more WRKY transcription factors were identified in other plants. For example, 116 WRKY transcription factors were identified in cotton and 133 were identified in soybean (Yin et al., 2013; Dou et al., 2014). These studies provide abundant nucleic acid resources for investigating important functions for the improvement of plants.

Many WRKY transcription factors have been cloned and functionally characterized. Through analysis of the *Atwrky6* mutant, *AtWRKY6* was found to be essential for root growth under low-boron conditions (Kasajima et al., 2010). In addition, it was considered as an arsenate-responsive transcription factor by mediating the expression of arsenate/phosphate transporter genes (Castrillo et al., 2013). *AtWRKY34* was found to express specially in the male gametophytes, and could negatively mediate cold sensitivity of pollen in *Arabidopsis* through the *CBF* signal cascade (Zou et al., 2010). Ding et al. (2014) demonstrated that overexpression of *AtWRKY46* lead to hypersensitivity to drought and salt stress by regulating the expression of *QUA-QUINE STARCH* (*QQS*). Allelic genes, *OsWRKY45-1* and *OsWRKY45-2*, are involved in different physiological processes. Overexpression of *OsWRKY45-2* resulted in hypersensitivity to ABA and the tolerance of transgenic plants to salt stress was reduced, whereas the transgenic plants with *OsWRKY45-1* overexpression showed an opposite phenotype (Tao et al., 2011). *OsWRKY42* was found to be up-regulated in senescent leaves and accelerated leaf senescence through the repression of *OsMT1d*-mediated scavenging of reactive oxygen species (Han et al., 2014). In addition, many other WRKY transcription factors were also found to be related to abiotic stress in different plants, such as soybean (Jaffar et al., 2016), barley (Meng and Wise, 2012) and *Thlaspi caerulescens* (Wei et al., 2008). All these studies prove that the WRKY transcription factors are involved in different physiological processes and are essential for abiotic stress responses in plants.

In wheat, 15 WRKY transcription factors (TaWRKYs) were cloned and their expression patterns were analyzed by Wu et al. (2008). Niu et al. (2012) assembled the expressed sequence tags (ESTs) of wheat present in NCBI and identified 43 TaWRKYs. They also reported that overexpression of *TaWRKY2* or *TaWRKY19* in *Arabidopsis* significantly improved the abiotic stress tolerance of the transgenic plants. As of date, only 76 TaWRKYs, including 40 TaWRKYs with full-length coding sequences (CDS), have been deposited in the NCBI database (Ma et al., 2014), and it significantly restricts the identification of TaWRKYs with important functions. In previous studies, we identified 78 WRKY transcription factors in *Triticum urartu* and 103 in *Aegilops tauschii* (Ma et al., 2014, 2015). On the basis of these 181 WRKY transcription factors, we identified 188 TaWRKYs in the present study. In addition, all of them were used for evolution studies. Furthermore, one TaWRKYs was found to be involved in regulating the stomatal closure to enhance the drought stress tolerance in transgenic *Arabidopsis* plants.

## Materials and Methods

### Plant Material and Treatments

The hexaploid wheat cultivar Aikang58, which is a typical wheat variety and is widely cultivated in the Huang-Huai-Hai Plain of China since 2003, was used in this study. The seeds of Aikang58 were sterilized and cultured to two-leaf stage, as described by Li et al. (2013). For inducing different degrees of osmotic stress, wheat seedlings were cultured in Hoagland solution containing different concentrations (0, 5, 10, 15, and 20%) of PEG-6000 (Sangon, Shanghai, China) for 24 h. For inducing abiotic stress at various time intervals, wheat seedlings were cultured in Hoagland solution containing 15% PEG-6000, 100 μmol/L ABA (Sangon, Shanghai, China) or 10 μmol/L H_2_O_2_ (Sangon, Shanghai, China) for 0, 6, 12, 18, and 24 h, respectively. The leaves and roots of wheat seedlings exposed to the different treatments were sampled and kept frozen at -80°C for subsequent qRT-PCR analysis and gene cloning.

### Database Searching for WRKY Transcription Factors in Hexaploid Wheat

We previously identified WRKY transcription factors form *T. urartu* and *A. tauschii* (Ma et al., 2014, 2015). The ancestors of both species are considered to be the progenitors of the A and D genomes of the hexaploid wheat genome (Jia et al., 2013; Ling et al., 2013). The CDS and protein databases of hexaploid wheat were downloaded according to the research of International Wheat Genome Sequencing Consortium (2014). The sequences of WRKY transcription factors from *T. urartu* and *A. tauschii*, representing different WRKY motif types, were used as queries to search against the CDS and protein sequences of hexaploid wheat using BLASTn and BLASTp, respectively. All similar sequences with an *E*-value cut-off of 1 × e^-5^ were considered to be the candidate WRKY transcription factors. The candidate WRKY proteins were confirmed by Pfam platform for Hidden Markov Model (HMM) searching (PF03106) with an *E*-value cut-off of 1 × e^-10^ (Finn et al., 2006). Subsequently, the remaining sequences with WRKY domain were further confirmed by SMART platform (SM000774) to identify the WRKY transcription factors in hexaploid wheat (Letunic et al., 2009).

### Phylogenetic Tree Construction and Duplication Analysis of TaWRKYs

Multiple sequence alignment of the amino acid sequences of the conserved WRKY domains was performed using ClustalW, and MEGA 5.10 software was used to construct an unrooted phylogenetic tree by the neighbor-joining method. Bootstrap analysis using 1,000 replicates was performed to assess the significance of each node.

Based on the results of BLASTn, two TaWRKYs, which exhibited more than 70% coverage, more than 70% identity, and only one duplication event, were considered to be the result of a duplication event (Wei et al., 2013; Ma et al., 2014). The number of non-synonymous (*K*_a_) substitutions and the number of synonymous (*K*_s_) substitutions were calculated according to the method of Nei and Gojobori (1986). The divergence time was calculated using the formula, *T* = *K*_s_/2*r*, where the synonymous mutation rate (*r*) was 6.56 × 10^-9^ (Gaut and Doebley, 1997).

### RNA Extraction and Expression Analysis of *TaWRKY146*

To analyze the expression patterns of *TaWRKY146*, total RNA was extracted using TRIzol reagent (Takara, Japan) according to the manufacturer’s instruction. First-strand cDNA was synthesized using SuperScript III reverse transcriptase (Invitrogen, United States). The qRT-PCR was carried out on ABI 7500 real-time PCR system (Applied Biosystems, United States), and the method of comparative 2^-ΔΔC_T_^ was used to calculate the relative expression levels of *TaWRKY146*.

### Cloning of *TaWRKY146*

The cDNA sequence of *TaWRKY146* was obtained from the genome database, and the specific primers (WF1 and WR1) were designed using the Oligo 6 software. After PCR amplification, a PCR product was obtained and cloned into pEASYTM-T1 cloning vector (TransGen, China) for sequencing (GENEWIZ, Beijing, China), and a 689-bp cDNA with an incomplete 3′-end was obtained. To obtain the full-length sequence of *TaWRKY146*, the 3′-end was further amplified with WF2 and WR2 primers using the SMARTer^TM^ RACE cDNA Amplification Kit (Clontech, Japan) according to the manufacturer’s instruction. The full-length cDNA was finally amplified using the WF3 and WR3 primers and sequenced by GENEWIZ (Beijing, China). All the primers and the CDS sequence are listed in Supplementary File 1.

### Overexpression of *TaWRKY146* in *Arabidopsis*

The CDS of *TaWRKY146* was inserted into a binary vector, pBI121, which contains a 35S promoter, and the recombinant plasmid was transformed into the *Agrobacterium tumefaciens* strain LBA4404. The *TaWRKY146* was transferred into *Arabidopsis* plants (Columbia Col-0) through *Agrobacterium*-mediated transformation using the floral dipping method (Clough and Bent, 1998). The positive transgenic lines were screened on 1/2 MS medium containing 50 μg/ml kanamycin (Solarbio, Beijing, China) and were confirmed by PCR amplification using the specific primers of *TaWRKY146*.

### Measurement of Malondialdehyde (MDA), Proline, and Soluble Sugar Contents

To determine the responses of the transgenic *Arabidopsis* plants to drought stress, the contents of MDA, proline and soluble sugar were measured according to the methods described by Bates et al. (1973) and Cui and Wang (2006).

## Results

### Identification of WRKY Transcription Factors in the Hexaploid Wheat

The CDS and protein sequences of WRKY transcription factors from *T. urartu* and *A. tauschii* were reported in our previous studies (Ma et al., 2014, 2015). The sequences of these WRKY transcription factors were used as queries in BLASTn and BLASTp searches for possible homologous sequences in the hexaploid wheat genome, which includes 111,982 expressed genes. After an extensive searching, a total of 818 candidate WRKY transcription factors were identified, and all the candidate WRKY protein sequences were further verified using Pfam and SMART platforms. Finally, 188 candidates were confirmed to be TaWRKYs, including 123 TaWRKYs with full-length CDS. The detailed information about these TaWRKYs, including their names, chromosome information, molecular weights, theoretical isoelectric points, CDS sequences and protein sequences, is provided in **Supplementary Data Sheet 2**.

Among the identified TaWRKYs, 48 were derived from the A genome, 53 from the B genome, and 47 from the D genome. And 40 TaWRKYs could not be located on chromosomes. We found that the TaWRKYs were distributed on seven chromosomes unevenly in the three genomes. Chromosomes 6 and 7 contained a few TaWRKYs, as in the case of *T. urartu* and *A. tauschii* (Ma et al., 2014, 2015), whereas chromosome 5 contained the highest density of TaWRKYs, with more than 10 TaWRKYs in all the three genomes (**Supplementary Data Sheet 2**).

### Phylogenetic and Evolutionary Analysis of TaWRKYs

**FIGURE 1:**
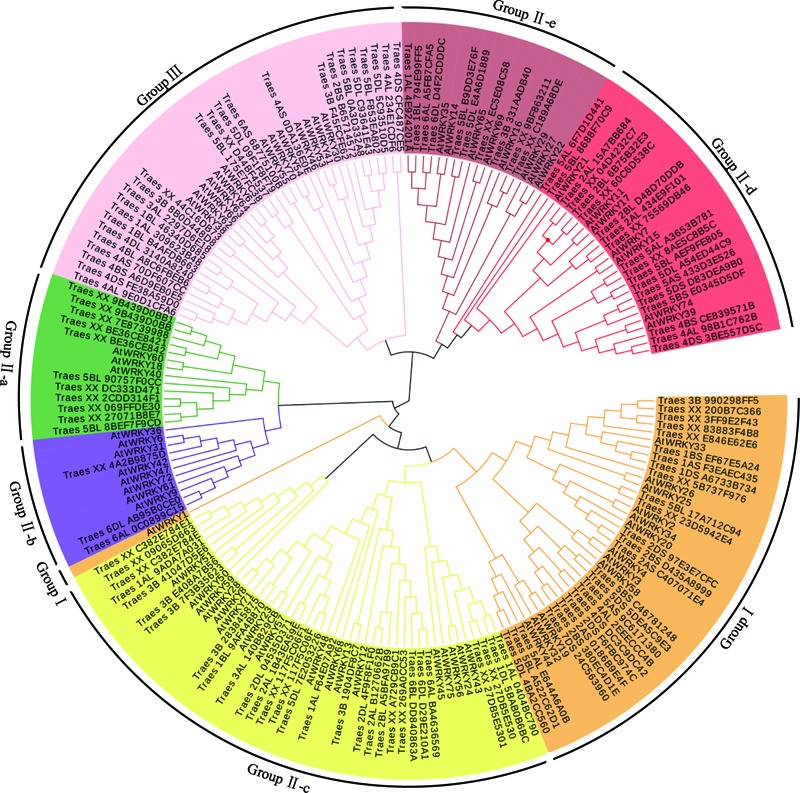
Phylogenetic tree analysis of WRKY transcription factors. The unrooted phylogenetic tree of the WRKY transcription factors in wheat and *Arabidopsis* was constructed by the neighbor-joining method using MEGA 5.10 software. The subgroups of the WRKY transcription factors are distinguished by different colors.

To examine the phylogenetic relationship of TaWRKYs with full-length CDS, an unrooted phylogenetic tree was constructed using the protein sequences of the WRKY domain from *Arabidopsis* and *T. aestivum* L. All the TaWRKYs were divided into three major groups: Groups I, II, and III containing 26, 72, and 25 TaWRKYs, respectively (**Figure 1**). Group II was further divided into five subgroups: Group II-a (11 TaWRKYs), Group II-b (3 TaWRKYs), Group II-c (29 TaWRKYs), Group II-d (19 TaWRKYs), and Group II-e (25 TaWRKYs), which was consistent with the previous observations in other plants. The proportion of WRKY transcription factors in the different groups was similar in wheat and *Arabidopsis* expect for Group II-a and Group II-b. In Group II-a, there were eleven TaWRKYs and three AtWRKYs, whereas there were three TaWRKYs and eight AtWRKYs in Group II-b. The proportion of WRKYs was reversed in the two species.

It was evident that the Group II-d, Group II-e, and Group III were located at the external of the phylogenetic tree, which indicated that the differentiation event of the ancestors of these TaWRKYs occurred earlier than those of the others resulting in their sequences being much different. Eleven clusters, containing three TaWRKYs that were derived from A, B, and D genomes, respectively, and were located on the same chromosomal arms in each genome, were observed. The phylogenetic tree further suggested that the A, B, and D genome progenitors of the hexaploid wheat genome should be closely related species. Based on this hypothesis, we speculated that *Traes_XX_4C16DB73, Traes_XX_D44BF4537*, and *Traes_XX_75569DB46* should be located on the chromosomal arms 3DL, 5DL, and 2DL, respectively. In these 14 clusters, TaWRKYs from the D genome appeared 12 times in the two internal branches, five times with TaWRKYs from the A genome and seven times with those from the B genome. These results indicate that the D genome should have more similarity with the A and B genomes and the latter two should have a relatively distant relationship.

### Gene Duplication Analysis of TaWRKYs

Of the 123 TaWRKYs with full-length CDS that were identified, 89 TaWRKYs could be located on chromosomes. 29 TaWRKYs were from the A genome, 36 TaWRKYs from the B genome, and 24 TaWRKYs from the D genome. As the progenitors of the A, B, and D genomes were closely related species, we analyzed the gene duplication individually in each of the genomes. Based on the parameters that had been used by Wei et al. (2013) and Ma et al. (2014), duplication events of four pairs of TaWRKYs appeared to have occurred, among which three pairs were derived from the B genome and one pair was from the D genome. A segmental duplication event was found to have occurred between *Traes_5BL_8688F70C9* and *Traes_2BL_6B75B32E3*. Other three pairs of TaWRKYs were found to be tandem duplication events (**Table 1**).

**Table 1:** Gene duplication analysis of TaWRKYs.

Gene pairs	*K*_s_	*K*_a_	*K*_a_/*K*_s_	Purifying selection	Duplication time (Mya)	Duplication type
Traes_5BL_0A3D332A8.1 Traes_5BL_F853EA802.1	0.3074	0.1886	0.6134	Y	23	Tandem
Traes_5BL_8688F70C9.1 Traes_2BL_6B75B32E3.1	0.8482	0.2525	0.2977	Y	65	Segmental
Traes_5BL_90757F0CC.1 Traes_5BL_8BEF7F9CD.1	0.2576	0.1079	0.4188	Y	20	Tandem
Traes_5DL_5C93510D5.1 Traes_5DL_C93641E43.1	0.2864	0.1951	0.6814	Y	22	Tandem

As suggested by Peng et al. (2012), positive selection should occur with *K*_a_/*K*_s_ > 1, neutral selection should occur with *K*_a_/*K*_s_ = 1, and purifying selection should occur with *K*_a_/*K*_s_ < 1. The *K*_a_/*K*_s_ ratios of the four pairs of TaWRKYs were all less than 1 (**Table 1**), indicating that purifying selection should have played a dominant role, and the detrimental variation should have been eliminated to maintain the gene function during the duplication process. The synonymous substitutions were used to calculate the duplication dates of the TaWRKY pairs. It showed that the duplication events of the four TaWRKY pairs took place between 20 and 65 million years ago (Mya) (**Table 1**).

### Expression Analysis of TaWRKYs in Different Tissues

In a previous study, 15 transcriptome profiles of wheat were generated using five tissues (grain, leaf, root, spike, and stem) from the beginning, middle and end stages (Choulet et al., 2014). The data are available in WheatExp^1^, and these data provide a platform for the analysis of the gene expression pattern in wheat. Of the 123 full-length TaWRKYs, 65 were found to be expressed in the five tissues. The results showed that most of the TaWRKYs were predominantly expressed in leaves and roots, and lower expression levels of some TaWRKYs were detected in grains at the middle-stage, leaves at the beginning-stage, spike at the end-stage and in stem (Supplementary File 1).

### Selection and Cloning of Abiotic-Related TaWRKY

**FIGURE 2:**
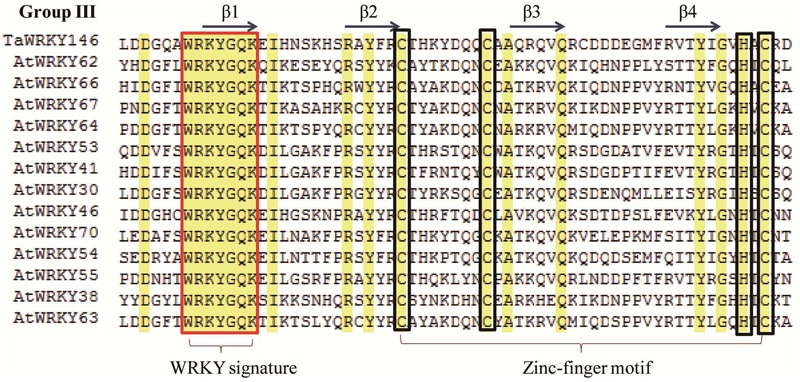
Multiple sequence alignment of the WRKY domains of *TaWRKY146* and AtWRKYs in Group III. The WRKY signature is highlighted in the red rectangular frame and the zinc finger motif is highlighted in the green rectangular frame.

In a previous study, we had identified the WRKY transcription factors, which are involved in the abiotic stress responses from *Arabidopsis* and rice (Ma et al., 2014). Herein, we determined the similarity between these abiotic stress-related WRKY transcription factors and TaWRKYs using BLASTp. *AtWRKY46*, which regulates osmotic stress responses in *Arabidopsis* (Ding et al., 2014), showed high similarity with *Traes_7DL_A9EF00572*. We, therefore, speculated that *Traes_7DL_A9EF00572* might be involved in osmotic stress responses. To further analyze the gene function, we first cloned the full-length CDS of *Traes_7DL_A9EF00572* through PCR amplification using the primers that were designed based on the sequence from the genome database. However, we obtained only a 689-bp cDNA sequence with an incomplete 3′-end. Consequently, the 3′-end was further amplified using the SMARTer^TM^ RACE cDNA Amplification Kit. Finally, the full-length CDS of *Traes_7DL_A9EF00572* with 897 nucleotides, encoding 299 amino acids, was cloned and its sequence was submitted to the GenBank (accession number: MF770640). This TaWRKY possesses one WRKY domain of 63 amino acids. The isoelectric point and molecular weight of *Traes_7DL_A9EF00572* were 7.45 and 25576.21, respectively. Based on its location on the chromosome, *Traes_7DL_A9EF00572* was named as *TaWRKY146*. It was classified into Group III based on the phylogenetic tree (**Figure 1**). From the multiple sequence alignment of the WRKY domains of *TaWRKY146* and AtWRKYs in Group III, it was obvious that the protein sequence of *TaWRKY146* contains one WRKYGQK domain and a C_2_H_2_ zinc finger motif (**Figure 2**).

### Expression Pattern of *TaWRKY146* under Different Abiotic Stress

**FIGURE 3:**
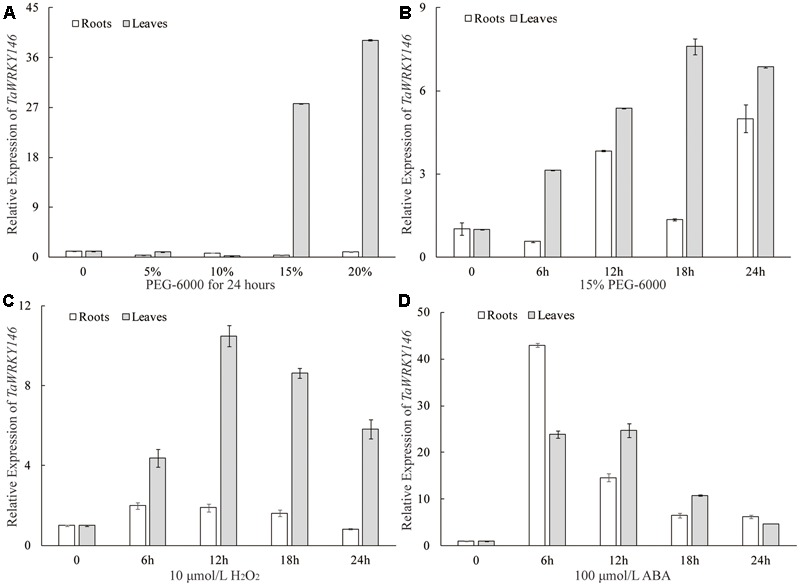
Expression patterns of *TaWRKY146* were examined in wheat seedlings under osmotic **(A,B)**, H_2_O_2_ **(C)** and ABA **(D)** treatments. Values are means ± SEs (*n* = 3).

The qRT-PCR was preliminarily used to determine whether the *TaWRKY146* was related to osmotic stress responses. We analyzed the expression patterns of *TaWRKY146* in the leaves and roots of wheat seedlings under different degrees of osmotic stress (0, 5, 10, 15, and 20% PEG-6000 for 24 h). We found that *TaWRKY146* was significantly up-regulated in the leaves under 15 and 20% PEG-6000 treatments, whereas the expression levels of *TaWRKY146* showed slight fluctuations in the roots (**Figure 3A**). Under osmotic stress, some response genes are induced to express instantaneously, some are stably expressed, and others are induced to express after exposure to stress for a long time. Therefore, the expression patterns of *TaWRKY146* in the leaves and roots of wheat seedlings, which were subjected to 15% PEG-6000 and 10 μmol/L H_2_O_2_ treatments for 0, 6, 12, 18, and 24 h, were also examined. *TaWRKY146* was found to be up-regulated to different levels. Overall, the expression levels in the leaves were higher than that in the roots (**Figures 3B,C**). Based on the expression patterns, we speculated that *TaWRKY146* might be closely related to osmotic stress.

### Overexpression of *TaWRKY146* in *Arabidopsis* Increases Drought Tolerance

**FIGURE 4:**
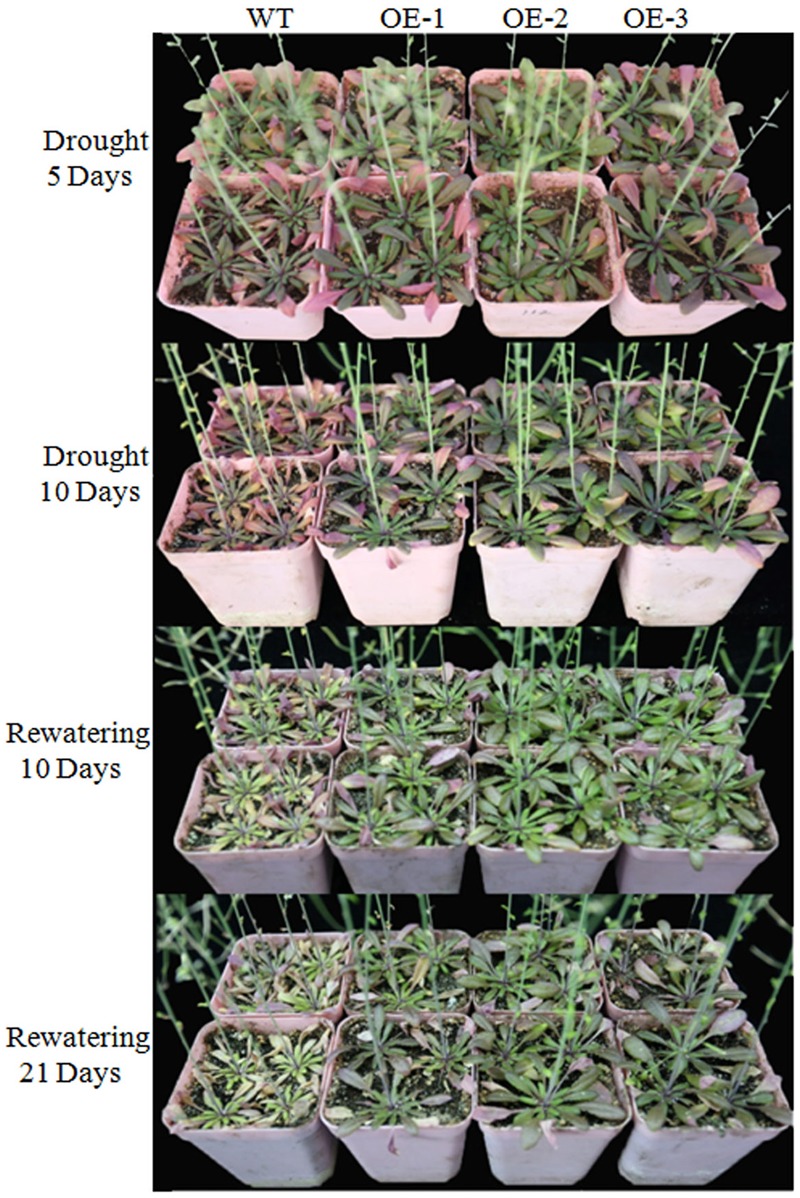
Phenotype of the wild-type plants and transgenic plants that were exposed to drought stress for 5 and 10 days and re-watered for 10 and 21 days during the bolting stage.

**FIGURE 5:**
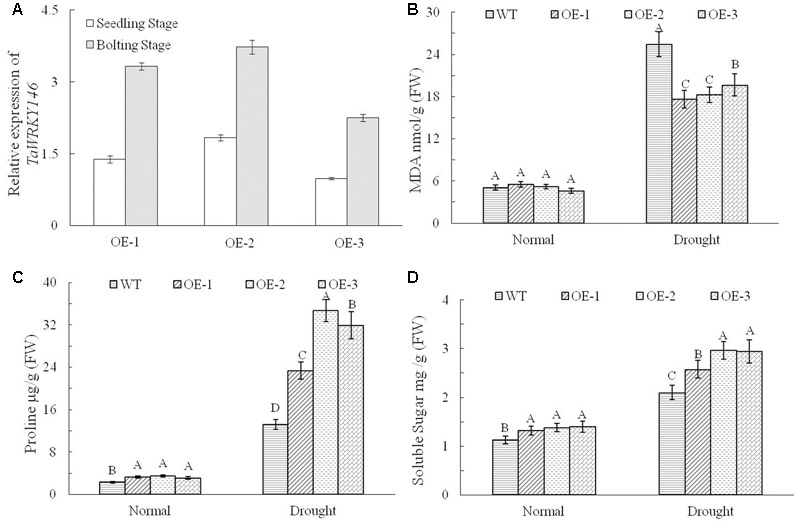
Expression levels of *TaWRKY146* were examined in the transgenic plants under drought stress at the seedling and bolting stages **(A)**. The contents of MDA, proline and soluble sugar were determined in the wild-type plants and transgenic plants under drought stress **(B–D)**. Three repetitions were measured for each line; the values are means ± SEs. The variance analysis was performed and the method of least significant difference (LSD) was used for multiple comparisons. The upper-case letters indicate the significant differences at 0.01 level.

We overexpressed *TaWRKY146* in *Arabidopsis* to investigate its function. Three transgenic lines (OE-1, OE-2, and OE-3) were generated. The plants of the three transgenic lines and the wild-type plants were subjected to drought stress by discontinuation of watering at the seedling and bolting stages, respectively. During the seedling stage, the transgenic plants were more resistant to drought stress compared to the wild-type plants (Supplementary File 1). During the bolting stage, the wild-type plants were severely dehydrated, whereas the plants of three transgenic lines were mildly dehydrated after 5 and 10 days of no watering. All the plants were re-watered on the eleventh day. After 10 and 20 days of re-watering, normal growth was resumed in the transgenic plants, whereas the leaves of the wild-type plants were all curled and exhibited the phenotype of severe dehydration (**Figure 4**). The expression levels of *TaWRKY146* were examined in the three transgenic lines at the seedling and bolting stages. *TaWRKY146* was expressed in all the three lines, and higher expression levels were observed at the bolting stage, especially in the OE-2 line (**Figure 5A**).

To investigate the responses of the transgenic plants and wild-type plants to drought stress, we determined the contents of MDA, proline and soluble sugar. No significant differences were observed between the transgenic plants and wild-type plants under normal growth conditions. However, there were significant differences in the contents of MDA, proline and soluble sugar between the transgenic plants and the wild-type plants exposed to drought stress (*p* < 0.01). Under drought stress, the content of MDA, which reflects to the extent of stress, was significantly higher in the wild-type plants than that in transgenic plants (**Figure 5B**), and the contents of proline and soluble sugar, which enhance the drought tolerance, were significantly higher in the transgenic plants (**Figures 5C,D**). All the above results indicated that overexpression of *TaWRKY146* in *Arabidopsis* could improve drought tolerance.

As the drought stress responses can occur through both ABA-dependent and ABA-independent pathways, we further examined the expression pattern of *TaWRKY146* in the leaves and roots of wheat seedlings, which were subjected to 100 μmol/L ABA for 0, 6, 12, 18, and 24 h. The results showed that *TaWRKY146* was sensitive to ABA and was found to be quickly up-regulated by exogenous ABA. The highest expression levels were observed at 6 or 12 h, and the expression levels in the leaves were higher than that in the roots (**Figure 3D**). Therefore, our results suggest that *TaWRKY146* should respond to drought stress through ABA-dependent pathway.

### *TaWRKY146* Induced Stomatal Closing under Drought Stress

**FIGURE 6:**
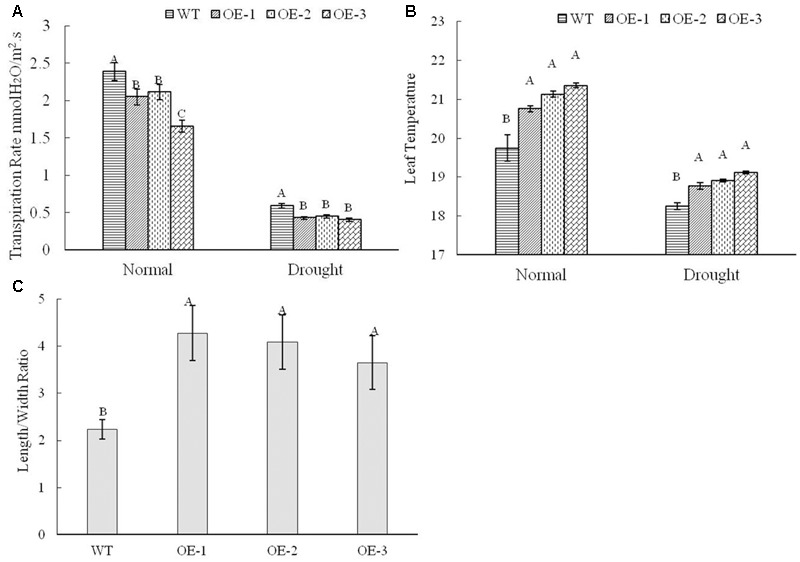
Transpiration rate **(A)** and leaf temperature **(B)** under normal and drought treatments were determined, and the length/width ratios of stoma **(C)** were obtained under drought stress in the wild-type plants and transgenic plants. Three repetitions were measured for each line; the values are means ± SEs. The variance analysis was performed and the method of LSD was used for multiple comparisons. The upper-case letters indicate the significant difference at 0.01 level.

Based on the phenotype, the plants with *TaWRKY146* overexpression showed higher tolerance to drought stress and had lower rate of water loss. *AtWRKY46*, which is a homologous gene of *TaWRKY146* with a bit score of 74 and an *E*-value of 9 × e^-14^ in the BLASTp search, was found to regulate osmotic stress via stomatal movement (Ding et al., 2014). Therefore, we speculated that the transgenic plants might differ from the wild-type plants in their stomatal closure. The transpiration rate and leaf temperature, which are related to water loss, were measured under normal and drought stress using Li-6400 (LI-COR, Lincoln, NE, United States) in the wild-type plants and transgenic plants. The transpiration rate was found to be higher in the wild-type plants compared to the transgenic plants, regardless of the normal and drought stress conditions (**Figure 6A**). As higher transpiration rate can dissipate more heat, higher leaf temperature was detected in the transgenic plants compared to that in the wild-type plants under normal and stress conditions (**Figure 6B**). It should be noted that overexpression of *TaWRKY146*, which should be expressed in transgenic lines under normal and stress conditions, might contribute to the reduction of evaporation rate.

**FIGURE 7:**
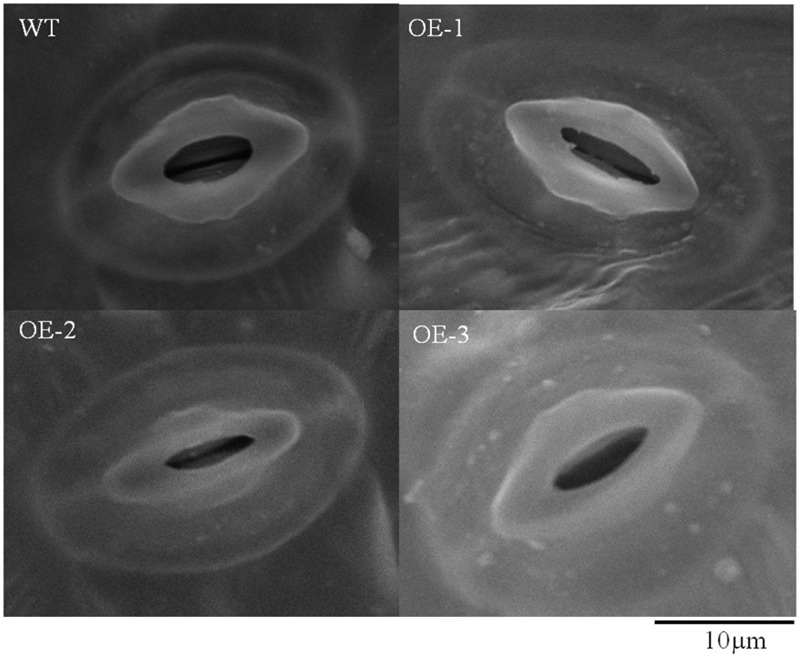
Stomatal aperture in the wild-type plants and transgenic plants that exposed to drought stress was observed using a scanning electron microscope.

Because there is a close relationship between the transpiration rate and stomatal closure, we determined the stomatal aperture of the wild-type plants and transgenic plants under drought stress using a scanning electron microscope. The results revealed that the stomatal opening was wider with smaller length/width ratio in the wild-type plants compared to that in the transgenic plants, and these differences were significant at 0.01 level (**Figures 6C, 7**). All these data indicated that overexpression of *TaWRKY146* could induce stomatal closure, thereby, reducing the water loss and increasing the drought tolerance.

## Discussion

As one of the most important transcription factor families, WRKY transcription factors are known to be involved in regulating the growth, phytohormone responses, and in improving abiotic and biotic stress tolerance. Because of their importance, the identification of these WRKY transcription factors have been already carried out from different plants, and the rapid development of whole-genome sequencing has greatly promoted these studies. In *Arabidopsis*, rice, cotton, and soybean, 72, 103, 116, and 133 WRKY transcription factors were identified, respectively, based on the sequence information present in the genome databases. Because of incomplete genome sequencing, only 15 and 43 TaWRKYs were identified using the wheat ESTs present in the NCBI database, respectively (Wu et al., 2008; Niu et al., 2012). Okay et al. (2014) identified 160 TaWRKYs using the amino acid sequences present in the Plant Transcription Factor Database v3.0 and the GenBank. Because of the large genome of wheat, more TaWRKYs needed to be identified. Moreover, the completion of whole-genome sequencing could provide important resource. A total of 188 TaWRKYs were finally identified in the present study, including 123 TaWRKYs with full-length CDS. This information should provide important resource for functional analysis. Previously, we had reported a total of 181 WRKY transcription factors from *T. urartu* and *A. tauschii*, which are the progenitors of the A and D genomes of the hexaploid wheat genome. There should be more than 200 WRKY transcription factors in the hexaploid wheat genome, which is much more than what is currently known. We believe that more TaWRKYs will be identified upon the completion of wheat genome sequencing.

The genes respond to drought stress mainly through the ABA-dependent and ABA-independent pathways (Yamaguchi-Shinozaki and Shinozaki, 2005). ABA is synthesized in the roots under drought stress and is further transferred to the leaves for the regulation of stomatal closure. In the present study, *TaWRKY146* was found to be mainly expressed in the leaves and relied on the ABA-dependent pathway to induce stomatal closure under drought stress. We speculated that the high concentration of ABA, which is induced by drought stress, could cause the up-regulation of *TaWRKY146*, which should further induce stomatal closure to retain water in plants and could, therefore, enhance drought tolerance.

Many WRKY transcription factors have been found to be related to abiotic stress. In previous studies, we have found 28 AtWRKYs and 13 OsWRKYs responding to abiotic stress and phytohormones by analyzing the other published reports (Ma et al., 2014). The *AtWRKY46*, which is a homologous gene of *TaWRKY146*, and *AtWRKY53* were found to positively mediate stomatal opening under drought stress (Ding et al., 2014; Sun and Yu, 2015). Herein, we found that *TaWRKY146* could induce stomatal closure under drought stress. Thus, WRKY transcription factors may respond to stress in different ways that need to be investigated in future research.

## Author Contributions

Conceived and designed the experiments: CL, JM, and LJ. Performed the experiments: JM, XG, QL, YS, and DZ. Wrote the paper: JM and XG. Edited the manuscript: JM.

## Conflict of Interest Statement

The authors declare that the research was conducted in the absence of any commercial or financial relationships that could be construed as a potential conflict of interest.

## Footnotes

1 http://wheat.pw.usda.gov/WheatExp/

## Supplementary Material

DATA SHEET 2 The names, chromosome information, molecular weights, theoretical isoelectric points, CDS sequences and protein sequences of TaWRKYs.
