# Supplementary material for: Overexpression of TaWRKY146 Increases Drought Tolerance through Inducing Stomatal Closure in Arabidopsis thaliana
Source: Front Plant Sci. 2017 Nov 24;8:2036. doi: 10.3389/fpls.2017.02036 (PMC5706409; doi:10.3389/fpls.2017.02036)
Supplement: Supplementary file 1 [file Data_Sheet_1.DOC]

**Supplemental Table 1** Primers used in this study.

| Primers | | | Sequences | | |  |  |  |
| --- | --- | --- | --- | --- | --- | --- | --- | --- |
| *TaWRKY146* | | |  |  |  |  |  |  |
|  | For the amplification of core sequence | | | |  |  |  |  |
|  |  | WF1 | 5'-ATGGAGGCCCCGTTGGCGCAG-3' | | | | | |
|  |  | WR1 | 5'-TCAGGCTTTATGCCCTGCAGG-3' | | | | | |
|  | For 3' RACE | |  |  |  |  |  |  |
|  |  | WF2 | 5'- GCAATCGTCCGTCGTAAC-3'  (3'race gene specific primer) | | | | | |
|  |  | WR2 | 5'–CTAATACGACTCACTATAGGGC-3'  (3' RACE universal primer) | | | | | |
|  | For full-length cDNA amplification | | | | | |  |  |
|  |  | WF3 | 5'-ATGGAGGCCCCGTTGGCGCAG-3' | | | | | |
|  |  | WR3 | 5'-TCAATGATCGAGTACGT-3' | | | | | |
|  | For qRT-PCR | | | | | | | |
|  |  | WF4 | 5'-CGGAGAAGGACCCAGCAATCG-3' | | | | | |
|  |  | WR4 | 5'-GCTCTCCATGCTCTTCGTT-3' | | | | | |

***Supplemental File 1. The full-length CDS of TaWRKY 146***

| >TaWRKY146 |
| --- |
| ATGGAGGCCCCGTTGGCGCAGGTGACGGATGACCTGATCAAGGGGCGGGAGTTGGCGACGCAGCTGCAGGGCCTCCTCCGGGACTCCCCCGAGGCCGGCGGTCTCATCGTGGACCAGATCCTCCACGCCTTCTCCCGCGCCATCCACGCCGCCAGGGCCGCGGCCGCTGCCAGCACCAGCGAGAGGTCCTCGGACGTGCGGAGCGAGGTCACCGACGGCGCGAGCGGCGGCGCGAAGAGGAAGTCCGCCTCCGCCGCCGGCGGAGGAAACCGCAGGGCCTGCCGGAGAAGGACCCAGCAATCGTCCGTCGTAACGAAGAGCATGGAGAGCTTGGACGACGGGCAGGCATGGCGCAAGTACGGGCAGAAGGAGATACACAACTCCAAGCACTCGAGGGCCTACTTCCGGTGCACGCACAAGTACGACCAGCAGTGCGCGGCGCAGCGGCAGGTCCAGCGCTGCGACGATGACGAGGGCATGTTCAGGGTCACCTACATCGGCGTGCACGCCTGCCGGGACCCCGCCGCCGCCGTGGCGCCGCACCTCCTCCACCACCTGAGCGGCGCCGCCCAAGGCCTGCACGCCGGCTGCCACCTCATCAGCTTCGCGCCCGGCAGCGTCGCTACCGCTACCCATGGCACCACCACCAGCACGGCGATGGGGTCCGGCCTGCAGGGCATAAAGCCTGAGAGCGGCGACCAGGAGGAGGTGCTGAGTAGCCGCACCCCCGGGAGCTCTGCCCTGCACAGCGCCGCCGCGGCGGCGACGGCGCCTACTTGGCCCGACCAGGGAGACGTGACGTCCACCCGGCAATACGGTGGCACCGTTAGCTTTGGAGAGTACCTTGATGACTATGCGTCCCTCGGGGACCTAGTGTCGTACGTACTCGATCATTGA |

**Supplemental Figures:**

**
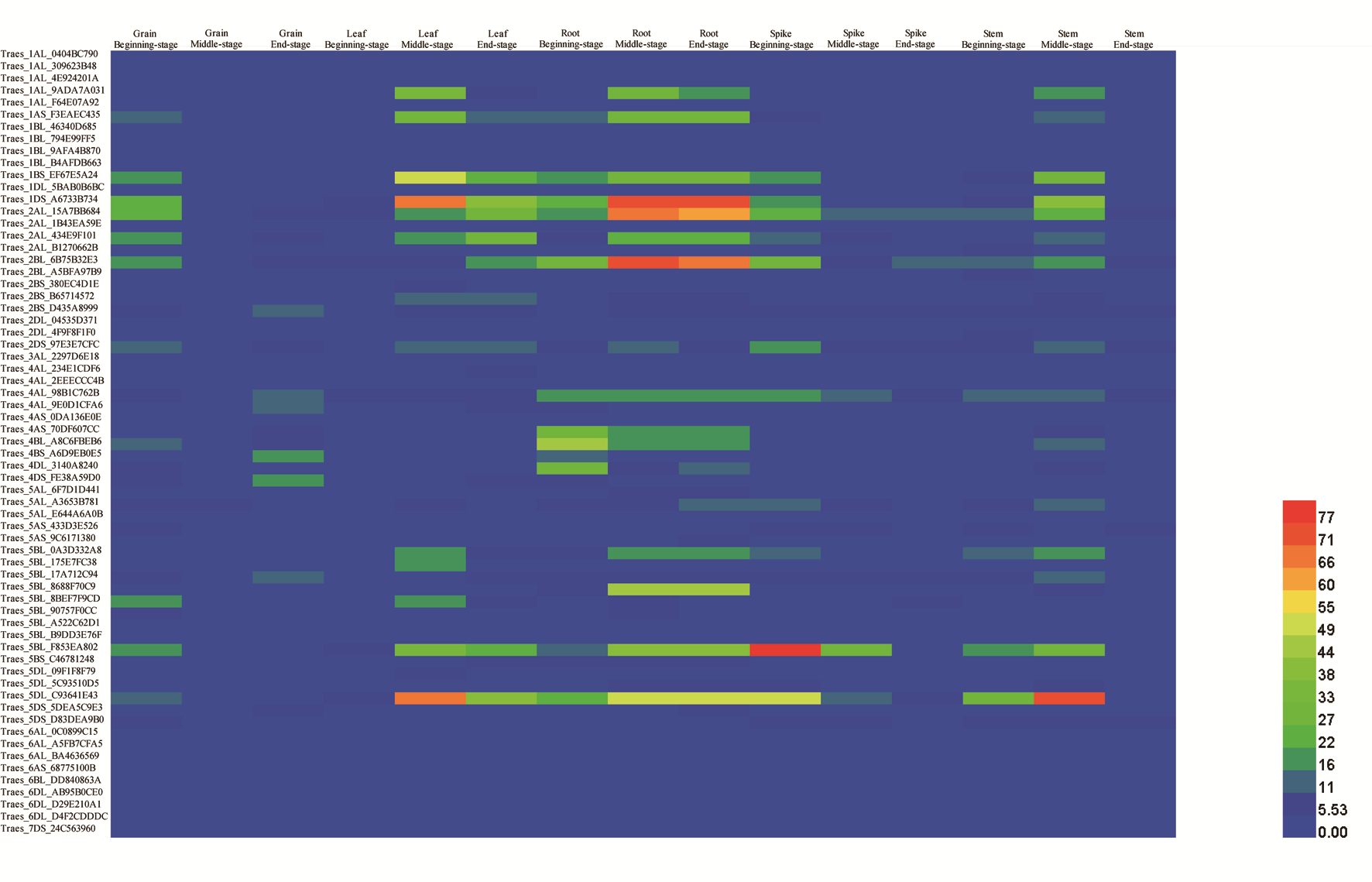
**

**Supplemental Figure 1. The expression pattern of TaWRKYs in the grains, leaves, roots, spikes and stems of wheat at the beginning, middle and end stages respectively.**

**
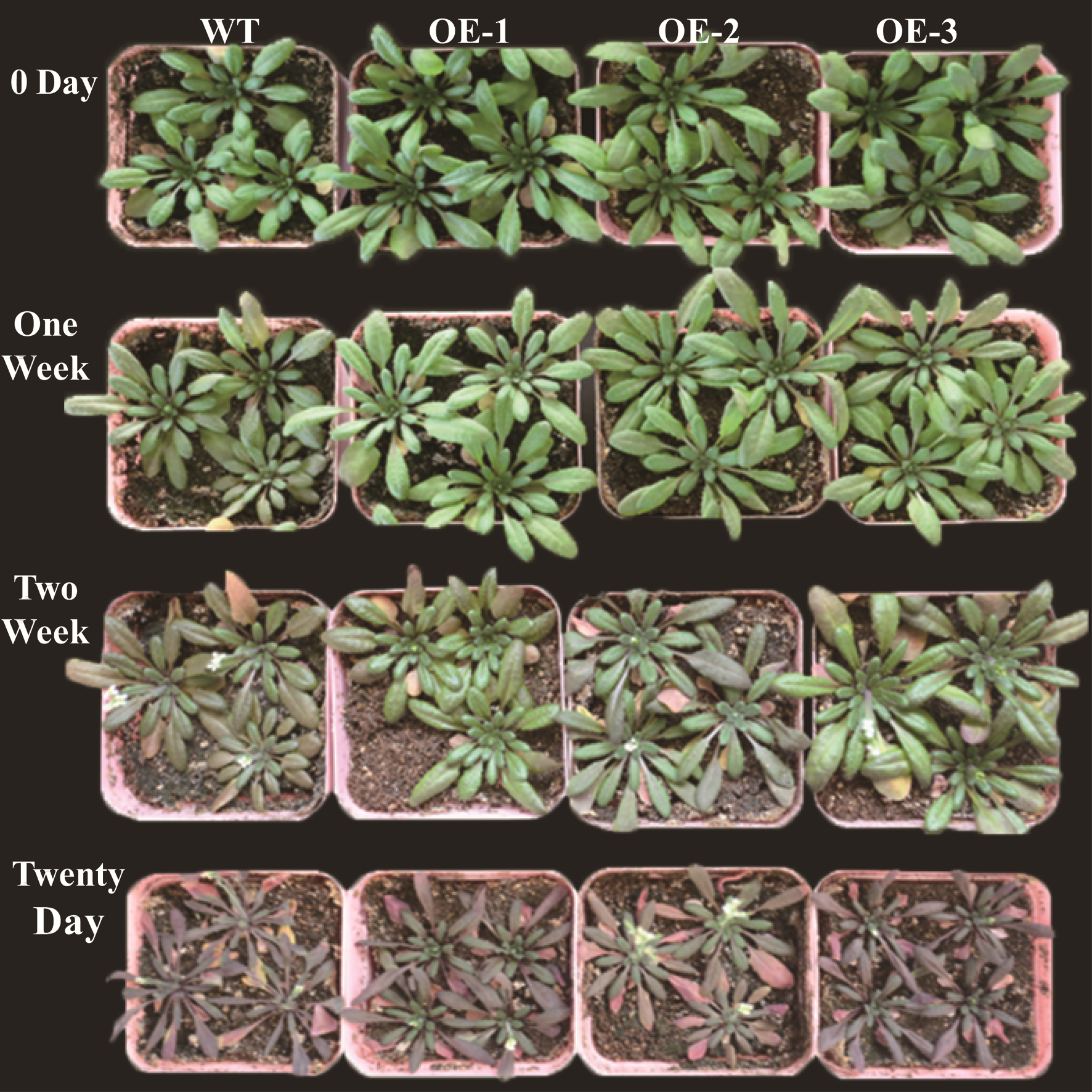
**

**Supplemental Figure 2. Phenotype of the wild-type and transgenic plants under drought stress for 0, 7, 14 and 20 days during the seedling stage.**
